# Recurrent Optic Perineuritis With Myelin Oligodendrocyte Glycoprotein Antibody-Associated Disease Complicated With Granulomatous Polyangiitis

**DOI:** 10.7759/cureus.25239

**Published:** 2022-05-23

**Authors:** Ken Nagahata, Shotaro Suzuki, Ritsuko Yokochi, Yuichiro Nei, Noboru Hagino

**Affiliations:** 1 Division of Hematology and Rheumatology, Teikyo University Chiba Medical Center, Ichihara, JPN

**Keywords:** tocilizumab, myelin oligodendrocyte glycoprotein antibody, optic perineuritis, anca-associated vasculitis, granulomatosis with polyangiitis

## Abstract

Optic perineuritis (OPN) is an intraorbital inflammatory disease that targets the optic nerve sheath, which can cause severe vision loss. OPN has been recently reported to be sometimes caused by myelin oligodendrocyte glycoprotein (MOG) antibody-associated disease (MOGAD). MOGAD is rarely reported to be complicated with other autoimmune diseases. We report the first rare case of MOG-associated OPN complicated with granulomatous with polyangiitis (GPA).

The vision loss, in this case, was initially considered to be caused by cavernous sinusitis in GPA. However, she was diagnosed with MOGAD with serial MRI findings and positive MOG antibody and had been successfully treated with glucocorticoid and tocilizumab for one and half years. This case emphasized the importance of evaluating the MOG antibody in a patient with recurrent OPN, complicated with vasculitis.

## Introduction

Optic perineuritis (OPN) is an intraorbital inflammatory disease that targets the optic nerve sheath; it is characterized by contrast-enhanced nerve sheath, tram-track, and donut-like findings on magnetic resonance imaging (MRI) [1]. The classic triad of OPN includes pain, optic neuropathy (relative afferent pupillary defect, loss of color vision, and contrast sensitivity), as well as swelling of the optic disc [2]. OPN has been recently reported to be associated with myelin oligodendrocyte glycoprotein (MOG) antibody-associated disease (MOGAD). MOGAD is an autoimmune disease of the central nervous system associated with a serological antibody against MOG, myelin oligodendrocyte glycoprotein. Relapsing vision loss due to optic neuritis/perineuritis is a typical feature of MOGAD; it can lead to severe vision loss. To date, coexisting systemic autoimmunity is not commonly observed in patients with MOGAD [3]. We report the first case of refractory MOGAD with granulomatosis with polyangiitis (GPA).

## Case presentation

**Figure 1:**
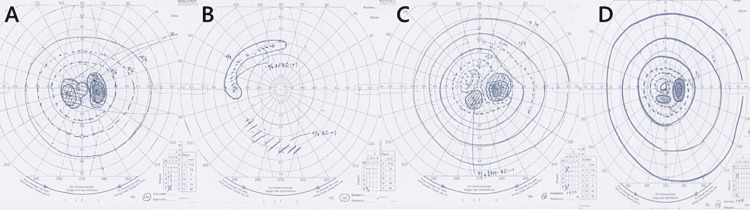
Funduscopic examination. (a) The right, large, dense central scotoma in the first attack. (b) A large visual field defect in the right eye during the second attack. (c) Right large central scotoma in the third attack. (d) Funduscopic examination of the right eye after recovery.

**Figure 2:**
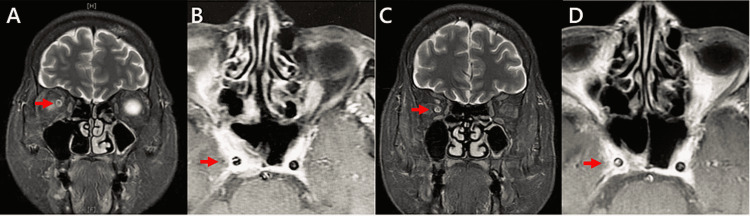
Magnetic resonance imaging. (a) A coronal view reveals a contrast-enhanced right optic nerve sheath of the first attack (arrow). (b) An axial view of T1-weighted MRI shows contrast-enhanced cavernous sinusitis of the attack (arrow). (c) A coronal view of third attack revealing a residual contrast effect around the right optic nerve (arrow). (d) An axial view of T1-weighted MRI showing a reduced contrast-enhanced of cavernous sinusitis of the third attack (arrow).

A 63-year-old female patient with no specific medical history presented with poor vision, redness, and pain in her right eye, as well as hearing loss on the right side over the previous six months. She had no fever, arthralgia, dyspnea, hemoptysis, or other neurological symptoms. Upon examination, her vital signs were found to be normal. She was alert and oriented and did not appear ill. The conjunctiva of the right eye was reddish with no exudate. No proptosis, eye deviation, or lacrimal enlargement was observed. Her heart sounds were normal, and her lungs were clear. The neurological history and cranial nerve examination were otherwise normal. On ophthalmologic examination, visual acuity in the left and right eye was 20/20 and 20/200, respectively. Visual field testing revealed a large, dense, right central scotoma (Figure 1a). Intraocular pressure was normal in both eyes. Funduscopic examination revealed no bleeding or ischemia. A diagnosis of right exudative otitis media and sinusitis was made after otolaryngology consultation. Laboratory tests revealed a white blood cell count of 10,800/μL, hemoglobin level of 12 g/dL, platelet count of 300,000/μL, C-reactive protein level of 4.2 mg/dL, and no electrolyte, liver, or renal function test abnormalities. The level of myeloperoxidase-antineutrophil cytoplasmic antibody (MPO-ANCA) was elevated (27 IU/mL, normal levels lee than 3.5 IU/mL). The test results for antinuclear antibody, anti-SS-A/SS-B antibodies, and PR3-ANCA were negative, as were the Treponema pallidum hemagglutination and rapid plasma reagin tests. No hematuria, proteinuria, or abnormal casts were observed on urinalysis. Head MRI with gadolinium enhancement showed cavernous sinusitis and a low contrast-enhanced right optic nerve sheath (Figures 2a, 2b), with no abnormal findings in the intraocular spaces and pachymeninges and no temporal artery thickening.

GPA was diagnosed based on elevated levels of MPO-ANCA, otitis media for more than three months, and retro-orbital inflammation [4]. Although right cavernous sinusitis to the right optic nerve or scleritis were considered possible causes of visual loss, the vision loss tentatively was presumably attributed to optic neuropathy. The combination of prednisolone (60 mg/day) and methotrexate (MTX) (10 mg/week), followed by intravenous methylprednisolone at a dose of 1 g/day for three days, resulted in rapid improvement in visual acuity. The dose of MTX was determined based on body weight and the domestic approved dose.

Twenty weeks after the induction therapy, while the patient was on prednisolone (9 mg/day) and MTX (10 mg/week), the visual field and light perception in her right eye gradually diminished over one week. Unlike in the initial attack, the patient reported no symptoms of scleritis. A relative afferent pupillary defect was present in the right eye, and other physical examination findings were normal. Visual field testing revealed an expansion defect (Figure 1b). C-reactive protein and MPO-ANCA levels were within the normal ranges. Neuromyelitis optica and multiple sclerosis were considered unlikely because there were no abnormal findings in the cerebrospinal fluid analysis, thoracolumbar spine MRI, and the test for anti-aquaporin 4 antibody. MRI showed a residual contrast effect around the right optic nerve. The positive findings of RAPD suggested that cavernous sinusitis might have caused OPN.

Following the administration of methylprednisolone (1 g/day for three days), the dose of prednisolone was increased to 60 mg/day and started rituximab (RTX) (500 mg/week for four weeks), resulting in rapid improvement of the visual acuity and field in the right eye.

Fourteen weeks later, while the patient was on prednisolone (10 mg/day), a third vision loss occurred in her right eye, with no signs of scleritis. Visual field examination revealed a large central scotoma (Figure 1c). MRI showed a contrast effect around the right optic nerve (Figure 2c), but cavernous sinusitis had disappeared (Figure 2d). No obvious paraneoplastic findings were observed through CT scan. A test for MOG antibody was conducted at the Tohoku University Department of Neurology, and the result was positive at a moderate level based on a cell-based assay.

The patient was diagnosed with MOG antibody-associated OPN. Intravenous TCZ (400 mg/week for four weeks) was adopted, followed by high-dose prednisolone. Her visual acuity and field recovered to the pre-onset level (Figure 1d). There was no recurrence of vision loss, and the prednisolone dose was tapered to 5 mg/day over the next 18 months with TCZ.

## Discussion

We report the first case of MOG antibody-associated recurrent OPN complicated with GPA. Her visual acuity improved to normal levels, and she was able to reduce her steroid dose with the combination of tocilizumab for 18 months.

This patient was classified as GPA by the European Medicines Agency algorithm [4]. Schirmer et al. reported that MPO-ANCA positive GPA had a clinical tendency of limited disease without severe organ involvement [5]. Furthermore, Sada et al. found that about half of GPA in Japan was MPO-ANCA positive [6]. Approximately 30% of patients with GPA present with ocular complications, such as scleritis, uveitis, intraorbital pseudotumor, retinal vasculitis, optic neuropathy, pachymeningitis, and nasolacrimal gland enlargement [7,8]. Optic neuropathy, a relatively uncommon symptom of GPA, can be attributed to compression mainly by retro-orbital tumors and cavernous sinusitis [9]. In this case, cavernous sinusitis was initially thought to be responsible for vision loss. At the third attack, though the cavernous sinusitis improved, OPN remained, and the MOG antibody was found to be positive, which led to the diagnosis of MOGAD.

MOGAD is an inflammatory disorder of the central nervous system characterized by immune-mediated demyelination attacks predominantly targeting the optic nerves, brain, and spinal cord. Optic neuritis, myelitis, brainstem encephalitis, and acute disseminated encephalomyelitis are frequently observed [10], but recurrent OPN occasionally has been reported [11]. As a treatment option, steroids and immunosuppressive drugs such as rituximab and methotrexate are commonly used [12], but for refractory cases, the efficacy of IL-6 inhibitors has recently been reported [13,14]. In this case, recurrent visual loss despite immunosuppressive therapies reminded us of the alternative diagnosis, MOGAD.

Coexisting systemic autoimmunity is not commonly observed in patients with MOGAD [3]. Among the 50 MOG antibody-associated cases, only one showed concomitant p-ANCA [15]; however, it was unclear whether this p-ANCA positivity was only serological or associated with clinically diagnosed ANCA associated vasculitis. Recently, Asano et al. reported the first case of MOGAD complicated with MPA [16], and this is the first case of MOGAD complicated with GPA.

Other possibilities than the coincidental complication of MOGAD and GPA, the onset of MOGAD may have been attributed to GPA. In this case, the clinical course of the initial attack accompanied by unilateral scleritis and elevated inflammatory responses and MPO-ANCA levels was very different from the second and third attacks without these findings. MOGAD sometimes occurs postinfection or postvaccination [17]. Bradl et al. pointed out that vaccination or infection may cause the activation of immune response and elevated cytokines, which could induce the breakdown of the blood-brain barrier and evoke MOG antibody pathogenesis [18]. In another report, Mitsuhashi et al. reported cerebrospinal fluid IL-6 levels in ANCA-associated vasculitis were elevated [19].

In this case, this hypothesis that immune response by GPA might have activated MOG antibody-specific B cells through increasing pro-inflammatory cytokines such as IL-6 seemed to be plausible. Still, it remained to be elucidated whether MOGAD-induced serial attacks of visual loss in this case independent of GPA because MOG antibody measurements of the initial vision loss could not be performed. Detailed studies of additional clinical cases are necessary to determine the causation.

## Conclusions

This is the first report of MOG antibody-associated OPN with GPA. Initially, vision loss was thought to be due to cavernous sinusitis in GPA, but MRI findings and MOG antibody helped diagnose MOGAD. Although the pathogenicity of ANCA in MOG is unclear, this case emphasized the importance of evaluating MOG antibodies in a patient with recurrent OPN, complicated with vasculitis.
